# Supplementary material for: D1- and D2-like receptors differentially mediate the effects of dopaminergic transmission on cost–benefit evaluation and motivation in monkeys
Source: PLoS Biol. 2021 Jul 1;19(7):e3001055. doi: 10.1371/journal.pbio.3001055 (PMC8248602; doi:10.1371/journal.pbio.3001055)
Supplement: S1 Table — a(cond) and e(cond) indicate the random effects of DAR blocking treatment conditions on parameters a and e, respectively. BIC is a relative measure of quality for the models (#1–4). ΔBIC denotes difference from minimum BIC. BIC, Bayesian information criterion; DAR, DA receptor. (PDF) [file pbio.3001055.s001.pdf]

D1R block (monkey KN)			
	model	BIC	ΔBIC
#1	*E = 1/a(cond)R*	-85.6	9.9
**#2**	***E = 1/a(cond)R + e***	**-95.5**	**0**
#3	*E = 1/aR + e(cond)*	-93.6	1.9
#4	*E = 1/a(cond)R + e(cond)*	-92.6	2.9

D1R block (monkey ST)			
	model	BIC	ΔBIC
**#1**	***E = 1/a(cond)R***	**-110.6**	**0**
#2	*E = 1/a(cond)R + e*	-98	12.6
#3	*E = 1/aR + e(cond)*	-92.4	18.2
#4	*E = 1/a(cond)R + e(cond)*	-95	15.6

D2R block (monkey KN)			
	model	BIC	ΔBIC
**#1**	***E = 1/a(cond)R***	**-126.7**	**0**
#2	*E = 1/a(cond)R + e*	-120.7	6
#3	*E = 1/aR + e(cond)*	-103.6	23.1
#4	*E = 1/a(cond)R + e(cond)*	-117.4	9.3

D2R block (monkey ST)			
	model	BIC	ΔBIC
**#1**	***E = 1/a(cond)R***	**-151.7**	**0**
#2	*E = 1/a(cond)R + e*	-138.8	12.9
#3	*E = 1/aR + e(cond)*	-124.2	27.5
#4	*E = 1/a(cond)R + e(cond)*	-135.4	16.3
