# Supplementary material for: D1- and D2-like receptors differentially mediate the effects of dopaminergic transmission on cost–benefit evaluation and motivation in monkeys
Source: PLoS Biol. 2021 Jul 1;19(7):e3001055. doi: 10.1371/journal.pbio.3001055 (PMC8248602; doi:10.1371/journal.pbio.3001055)
Supplement: S2 Table — (Rt|*) indicates random effects on regression parameters. E, refusal rate; Rt, reaction time; cond, treatment condition; monkey, subject. DAR, DA receptor. (PDF) [file pbio.3001055.s002.pdf]

Model		D1 block		D2 block	
		BIC	ΔBIC	BIC	ΔBIC
#1	*E ~ Rt*	**-1476.2**	**0**	**-1000.5**	**0**
#2	*E ~ Rt + (Rt|monkey)*	-1435.1	41.1	-988.1	12.4
#3	*E ~ Rt + (Rt|cond)*	-1443.4	32.8	-964.9	35.6
#4	*E ~ Rt + (Rt|monkey) + (Rt|cond)*	-1437	39.2	-980.9	19.6
