# Supplementary material for: D1- and D2-like receptors differentially mediate the effects of dopaminergic transmission on cost–benefit evaluation and motivation in monkeys
Source: PLoS Biol. 2021 Jul 1;19(7):e3001055. doi: 10.1371/journal.pbio.3001055 (PMC8248602; doi:10.1371/journal.pbio.3001055)
Supplement: S3 Table — CU and E0 indicate remaining cost and intercept, respectively. (0+ CU|*) and (CU|*) indicate random effects on both regression coefficient and intercept (E0) or on regression coefficient alone, respectively. E, refusal rate; type, trial type (delay or work); cond, treatment condition (CON, MO, and HO for D1R and D2R blocking); monkey, subject. CON, control; D1R, D1-like receptor; D2R, D2-like receptor; DAR, DA receptor; HO, high occupancy; MO, moderate occupancy. (PDF) [file pbio.3001055.s003.pdf]

	model	BIC	ΔBIC
#1	$E \sim RC + E_0 + (0 + CU\|type)$	**4405.2**	**0**
#3	$E \sim RC + E_0 + (0 + CU\|type) + (CU\|monkey)$	4621.5	216.3
#2	$E \sim RC + E_0 + (0 + CU\|type) + (CU\|cond)$	4745.1	339.8
#4	$E \sim RC + E_0 + (0 + CU\|type) + (CU\|monkey) + (CU\|cond)$	4858.1	452.9
