# Supplementary material for: D1- and D2-like receptors differentially mediate the effects of dopaminergic transmission on cost–benefit evaluation and motivation in monkeys
Source: PLoS Biol. 2021 Jul 1;19(7):e3001055. doi: 10.1371/journal.pbio.3001055 (PMC8248602; doi:10.1371/journal.pbio.3001055)
Supplement: S4 Table — (Rt|*) indicates random effects on regression parameters. E, refusal rate; Rt, reaction time; cond, treatment condition; monkey, subject. DAR, DA receptor. (PDF) [file pbio.3001055.s004.pdf]

	model	Delay		Workload	
		BIC	ΔBIC	BIC	ΔBIC
**D1R block**					
#1	*E ~ Rt*	1387.1	81.4	**1308.2**	**0**
#2	*E ~ Rt + (Rt|monkey)*	**1305.7**	**0**	1308.6	0.4
#3	*E ~ Rt + (Rt|cond)*	1374.1	68.4	1331.3	23.2
#4	*E ~ Rt + (Rt|monkey) + (RT|cond)*	1329.3	23.5	1324.4	16.3

	model	Delay		Workload	
		BIC	ΔBIC	BIC	ΔBIC
**D2R block**					
#1	*E ~ Rt*	1114.8	38.3	1228.9	72.6
#2	*E ~ Rt + (Rt|monkey)*	**1076.5**	**0**	1186.2	30
#3	*E ~ Rt + (Rt|cond)*	1090.7	14.3	1191.0	34.7
#4	*E ~ Rt + (Rt|monkey) + (RT|cond)*	1080.1	3.6	**1156.3**	**0**
