# Supplementary material for: D1- and D2-like receptors differentially mediate the effects of dopaminergic transmission on cost–benefit evaluation and motivation in monkeys
Source: PLoS Biol. 2021 Jul 1;19(7):e3001055. doi: 10.1371/journal.pbio.3001055 (PMC8248602; doi:10.1371/journal.pbio.3001055)
Supplement: S5 Table — (Rt|*) indicates random effects on regression coefficient. E, refusal rate; Rt, reaction time; cond, treatment condition; monkey, subject. D1R, D1-like receptor; D2R, D2-like receptor. (PDF) [file pbio.3001055.s005.pdf]

model		Delay		Workload	
		BIC	ΔBIC	BIC	ΔBIC
#1	*E ~ Rt*	**1262.2**	**0**	1390.4	41.2
#2	*E ~ Rt + (Rt|monkey)*	1276.8	14.6	1392.4	43.2
#3	*E ~ Rt + (Rt|cond)*	1266.5	4.3	**1349.2**	**0**
#4	*E ~ Rt + (Rt|monkey) + (Rt|cond)*	1280.1	17.9	1352.5	3.3
