# Supplementary figures and images for: D1- and D2-like receptors differentially mediate the effects of dopaminergic transmission on cost–benefit evaluation and motivation in monkeys
Source: PLoS Biol. 2021 Jul 1;19(7):e3001055. doi: 10.1371/journal.pbio.3001055 (PMC8248602; doi:10.1371/journal.pbio.3001055)

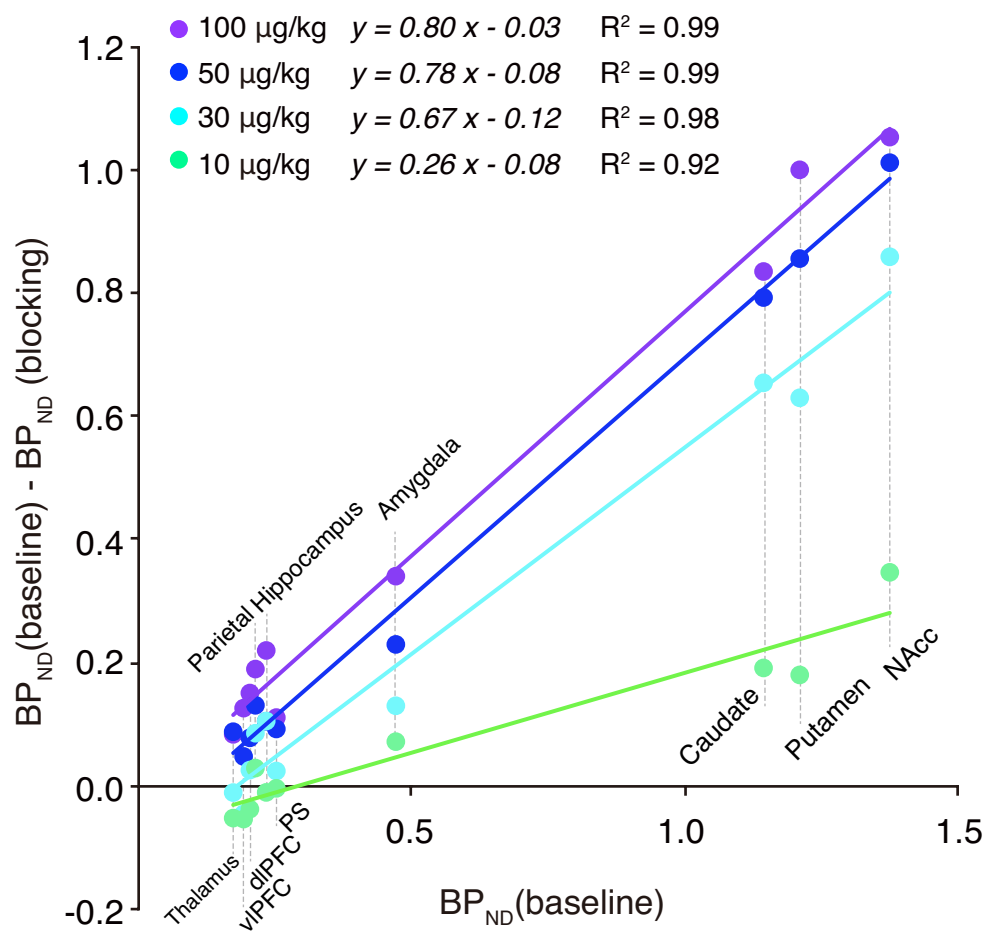

Supplement: S1 Fig — Example of occupancy estimation based on modified Lassen plot of [11C]SCH23390 PET data obtained from monkey DO. Colored dots represent the relationship between decreased specific binding [i.e., BPND (baseline)–BPND (blocking)] and baseline [BPND (baseline)] for each brain region under each blocking condition (indexed by color). Occupancy was determined as a proportion of reduced specific binding to baseline, which corresponds to the slope of linear regression. In this case, D1 occupancy was 80%, 78%, 67%, and 26% for 100, 50, 30, and 10 μg/kg doses, respectively. The data underlying this figure can be found on the following public repository: https://github.com/minamimoto-lab/2021-Hori-DAR. PET, positron emission tomography. (PDF) [file pbio.3001055.s006.pdf]

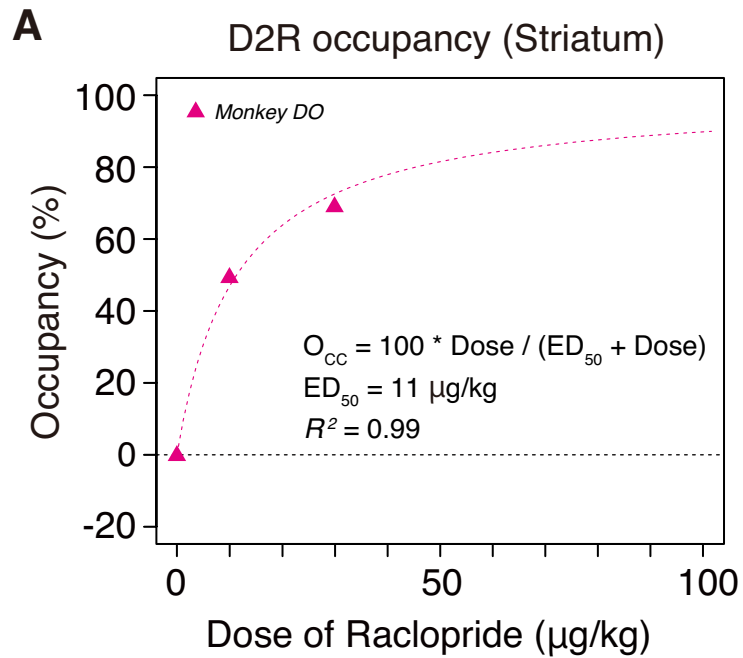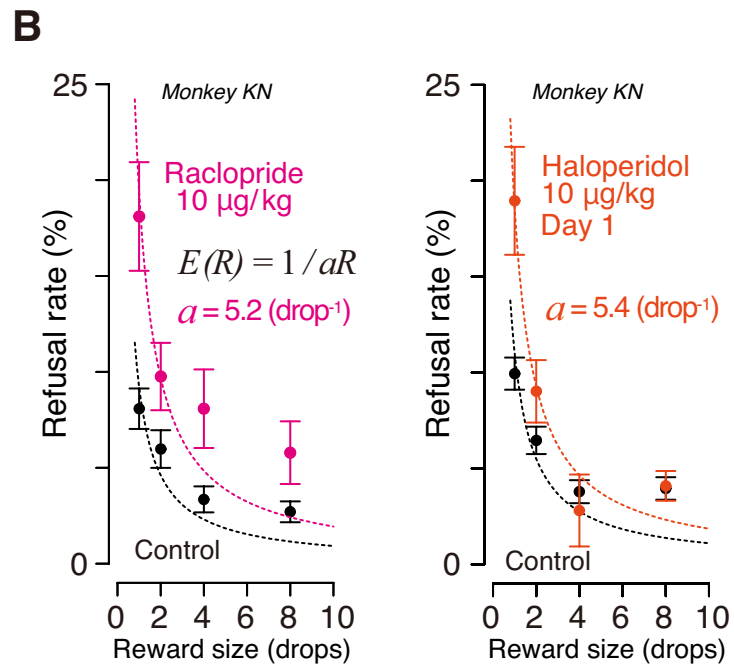

Supplement: S2 Fig — (A) Occupancy of D2R measured at striatal ROI is plotted against dose of raclopride. (B) Error rates as a function of reward size for control (black) and after injection of raclopride (10 μg/kg, i.m. left side) and haloperidol (10 μg/kg, i.m. right side) in monkey KN are plotted. Dotted curves are best-fit inverse function (model #1 in S1 Table). The data underlying this figure can be found on the following public repository: https://github.com/minamimoto-lab/2021-Hori-DAR. D2R, D2-like receptor; ROI, region of interest. (PDF) [file pbio.3001055.s007.pdf]

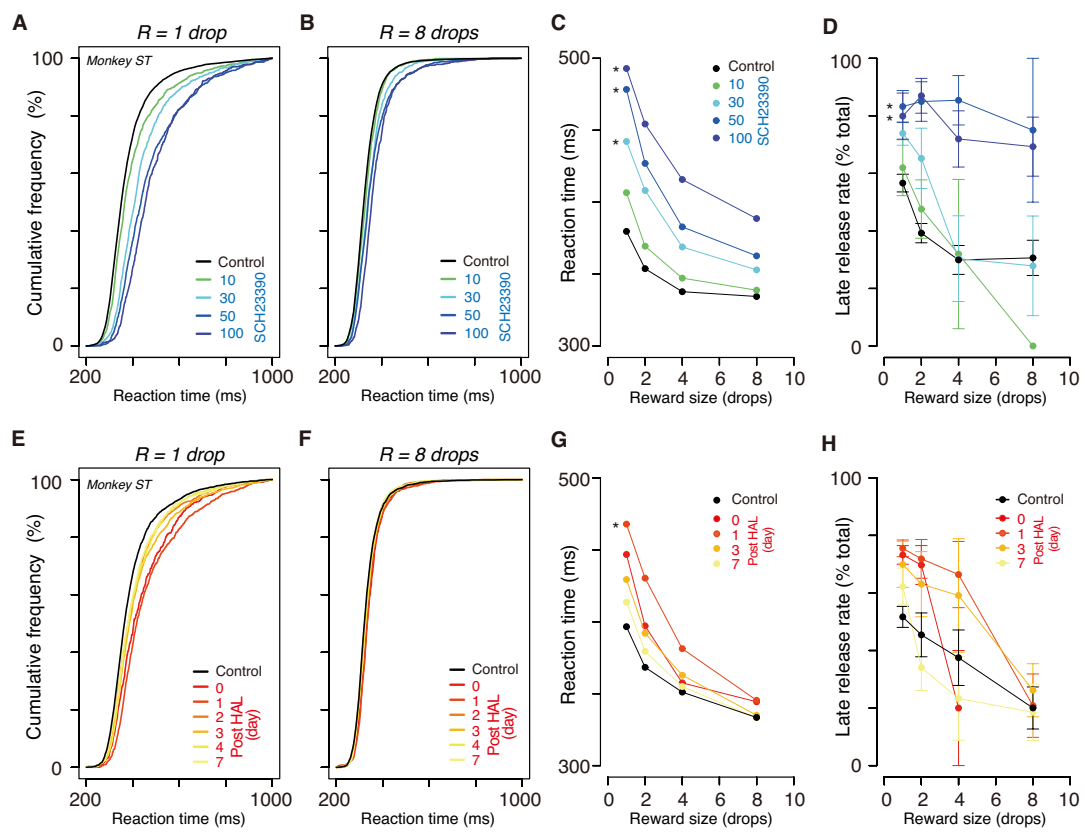

Supplement: S3 Fig — (A, B) Cumulative distribution of RT for control and D1R blockade conditions in drop-1 and drop-8 trials, respectively. (C) Mean RT as function of reward size for control and D1R blockade conditions. Two-way ANOVA, reward × condition; main effect of condition, F(4, 164) = 109.8, p < 1.0 × 10^−15^; main effect of reward, F(3, 164) = 111.0, p < 10−15; interaction, F(12, 164) = 4.7, p < 1.0 × 10^−5^. (D) Late release rate (mean ± SEM) as function of reward size for control and D1R blockade conditions. Two-way ANOVA, reward × condition; main effect of condition, F(4, 163) = 18.6, p < 1.0 × 10^−11^; main effect of reward, F(3, 163) = 9.8, p < 10−5; interaction, F(12, 163) = 1.0, p = 0.4. (E–H) Same as (A–D), but for D2R blockade. RT; main effect of condition, F(6, 92) = 7.2, p < 1.0 × 10^−5^; main effect of reward, F(3, 92) = 81.9, p < 10−15; interaction, F(18, 164) = 0.6, p = 0.65. Late release rate; main effect of condition, F(6, 90) = 3.5, p = 0.0038; main effect of reward, F(3, 90) = 19.2, p < 10−9; interaction, F(18, 90) = 1.4, p = 0.14. * significantly different from control, p < 0.05 post hoc Tukey HSD. Data were obtained from monkey ST. The data underlying this figure can be found on the following public repository: https://github.com/minamimoto-lab/2021-Hori-DAR. D1R, D1-like receptor; D2R, D2-like receptor; HSD, honestly significant difference; RT, reaction time. (PDF) [file pbio.3001055.s008.pdf]

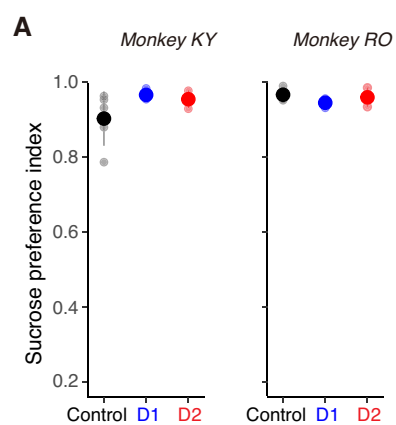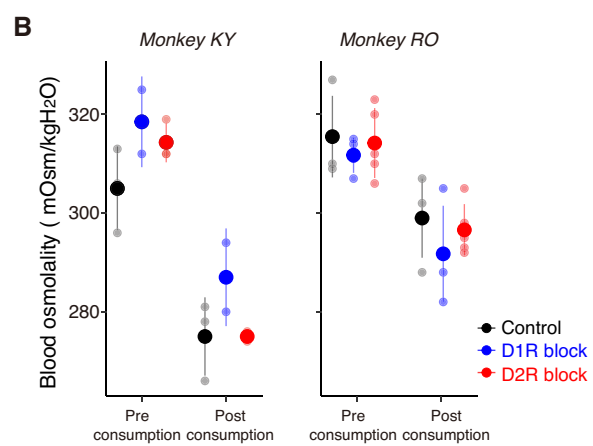

Supplement: S4 Fig — (A) Sucrose preference index after administration of saline (Control), SCH23390 (30μg/kg, D1), and haloperidol (10μg/kg; D2, day 0), respectively. There was no significant effect of DAR blockade on overall intake (1-way ANOVA, treatment, monkey KY, F(2, 8) = 1.26, p = 0.33; monkey RO, F(2, 14) = 2.01, p = 0.17) or sucrose preference (1-way ANOVA; treatment, monkey KY, F(2, 8) = 1.62, p = 0.26; monkey RO, F(2, 8) = 1.38, p = 0.31). (B) Blood osmolality measured in serum samples obtained before (pre) and after (post) sucrose test. There was no significant impact of DAR blockade (2-way ANOVA, monkey KY, main effect of treatment, F(2, 10) = 4.0, p = 0.056; pre-post, F(1, 10) = 93.83, p = 2.1 × 10^−6^, interaction, F(2,10) = 0.74, p = 0.50; monkey RO, treatment, F(2, 20) = 1.22, p = 0.32; pre-post, F(1, 20) = 40.8, p = 3.1 × 10^−6^, interaction, F(2,20) = 0.13, p = 0.88). Filled circles and shades indicate median and raw data points, while horizontal bars indicate SD. The data underlying this figure can be found on the following public repository: https://github.com/minamimoto-lab/2021-Hori-DAR. DAR, DA receptor. (PDF) [file pbio.3001055.s009.pdf]

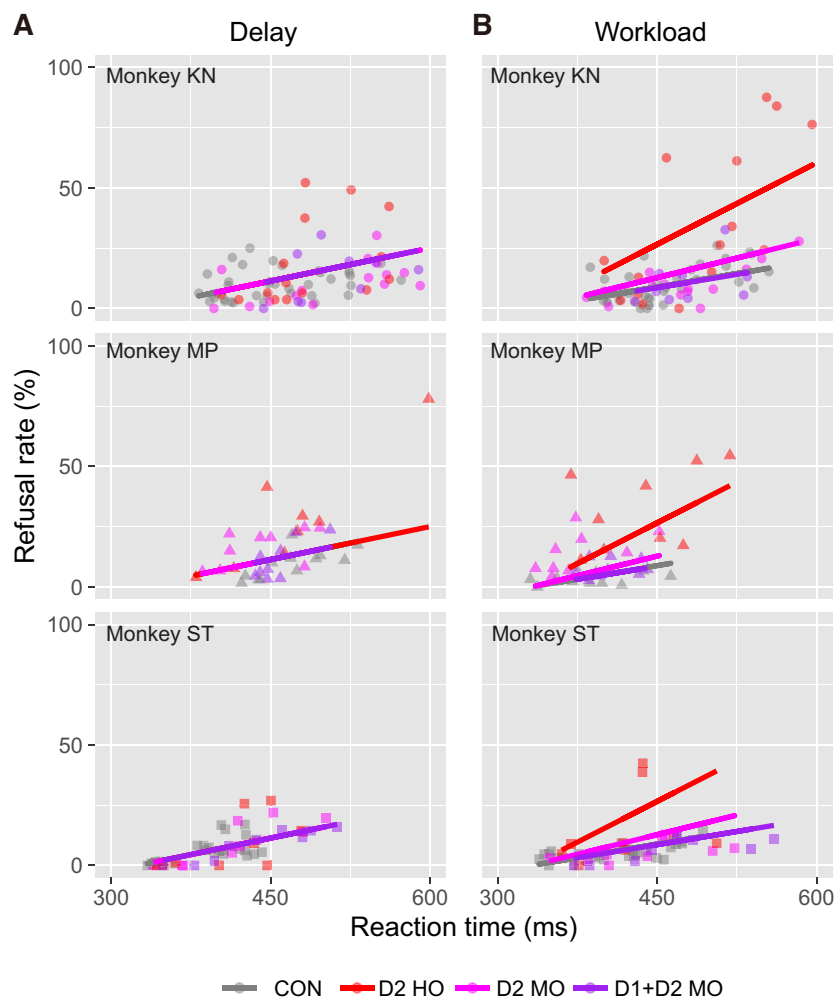

Supplement: S5 Fig — (A) Relationship between refusal rate and average RT for each reward size in session by session for D2 blocking and D1+D2 blocking in delay trials. Data are plotted individually for monkeys KN, MP, and ST, in order from top to bottom. Colors indicate treatment condition. Thick lines indicate linear regression lines (model #1 in S5 Table). (B) Same as A, but for workload trials. Note that for the data in workload trials, a multiple linear model with random effect of condition (model #3 in S5 Table) was chosen as the best model to explain the data, where the steepness of the slope under D1+D2 treatment was the same as that of control. The data underlying this figure can be found on the following public repository: https://github.com/minamimoto-lab/2021-Hori-DAR. D1R, D1-like receptor; D2R, D2-like receptor; RT, reaction time. (PDF) [file pbio.3001055.s010.pdf]
