# Supplementary material for: D1- and D2-like receptors differentially mediate the effects of dopaminergic transmission on cost–benefit evaluation and motivation in monkeys
Source: PLoS Biol. 2021 Jul 1;19(7):e3001055. doi: 10.1371/journal.pbio.3001055 (PMC8248602; doi:10.1371/journal.pbio.3001055)
Supplement: Attachment — Submitted filename: response0405.docx [file pbio.3001055.s011.docx]

***Reviewer #1****: This is the first study to directly compare the contribution of the dopamine D1R and D2R by showing the degree of binding to the striatum and other brain structures. I think the series of results that the authors presented are very convincing and the experimental procedure of the authors is straightforward and excellent. I have a few following concerns. If the authors revise the manuscript considering the following comments, the paper will be much improved. The task paradigm and the obtained change in behaviors are very convincing because of the established procedure of the author's series of experiments. However, for the readers who are not familiar with the task procedures, it is better to add a detailed explanation of the task and add further interpretation of the results. For example, each assignment and the experimental process is accurately described, but the conclusions are drawn from them, and their interpretation is not emphasized. As a result, each assignment's significance and its importance to the reader is often not conveyed to the reader.*
R: Thank you for the positive and constructive comments.

*Major comments:
(1)     Regarding the data in Figure 1, it is very important to examine the degree of D1R and D2R block by examining the occupancy with PET, and I think it is clean data. However, I fear that long-term plastic changes may be a side effect, especially in D2R, because of the long-lasting occupancy. The possibility of mixed plastic changes caused by long-term blocks needs to be discussed in detail.*

R: According to the reviewer’s advice, we added the following discussion regarding the possibility of plastic change due to long-term blocks in *Limitations of the current study* section in the Discussion:

“Haloperidol induced long-lasting occupancy for several days, thereby potentially causing unexpected long-term changes, such as synaptic plasticity. Although we cannot eliminate the potential effects of plastic change, a comparable behavioral impact was also observed after raclopride administration, which would induce short-term occupancy (cf. S2 Fig), supporting the view that blockade of DAR reduced motivation in an occupancy dependent manner.” (Line 511-516)

*(2)     On the relationship between reaction time and refusal rate, at L201, the authors mentioned, "D1R blockade also influences response speed, probably due to slowing cognitive processing". If the motivation itself is decreased by D1R block, it may directly affect reaction time and refusal rate. In this case, it is not necessarily related to whether there is some disorder to cognitive processing or not. The interpretation of this behavior, especially the relationship between RT and the refusal rate, needs to be discussed in detail.*
R: We agree with the reviewer that there was some confusion between the terms cognition and motivation. In our framework, motivation is a hidden process which affects behavior as a function of expected outcome value. It may or may not be considered as a cognitive process, but that is essentially a semantic issue. We have rewritten several sections of the manuscript to clarify this. More specifically, in that framework, as suggested by the reviewer, the influence of DAR manipulation on RT and refusal rate could be accounted for by a modification of a single computational variable: motivation, and therefore be correlated. We examined this possibility by analyzing whether RTs and refusal rates correlated session-by-session and whether and how this relationship was modified by DAR blockade. We found that they were linearly related consistently across D1 and D2 antagonism conditions. These results suggest that, as the reviewer pointed out, DAR antagonism may influence a single process (motivation) which affects both reaction time and refusal rate.

More importantly, similar linear relations were also observed in the data obtained with the work/delay task except for work trials in the D2R blockade sessions, where the slope of linear regression was significantly steeper along with occupancy. These results provide additional evidence to support the differential contributions of D1R and D2R on cost-benefit decision-making. Given the importance of these results, we have clarified this conceptual relationship between RT and refusal rate in the main Figs. 4, 6, and S5 Fig., and reported it with interpretation in the Results.

*(3)     "Relative reward" in L213-215 is an abrupt concept that needs to be explained; what is the purpose of the Sucrose preference test?*
R: We rephrased the section title and added an explanation about the purpose of the sucrose preference test in the Results, as follows:

“**Little influence of D_1_R- or D_2_R-blockade on hedonic impact of reward**

The behavioral data shown above suggest that blockade of DAR attenuates the incentive effect of reward on behavior. To evaluate the impact of DAR blockade on other aspects of motivation and reward processing, we also examined the effect of DAR manipulation on hedonic processes, i.e., on how pleasant was the reward consumption. In line with previous experiments in rodents [15, 50], we did not find any effect of treatment with D_1_R or D_2_R antagonist on overall intake or sucrose preference in either of the 2 monkeys tested (S4A Fig; see legend).” (line 245-251)

*(4)     About L300-302, the authors introduced concepts of "delay discounting" and "workload discounting." Although the relationship between them was carefully discussed in the Discussion, I would appreciate it if the authors add detail conceptual details when they introduce them (i.e., at Results).  At least, the author should add "why" they focused on these parameters.*

R: Delay- and workload-discounting simply refer to standard experimental procedures that manipulate cost, and therefore decrease motivation. This is now explained in the last paragraph of the Introduction. We also included a more detailed explanation of delay- and workload-discounting and the rationale for testing the corresponding model parameters in the Results as follows:

“The trade-off between the reward and costs of obtaining the reward affects decision-making as well as motivation. Both humans and animals have the tendency to prefer immediate, smaller rewards over larger, but delayed rewards. The preference can be predicted by discounting the reward’s intrinsic value by the duration of the expected delay, an effect designated as “delay discounting” [52, 53]. Discounting of the reward value also occurs in proportion to the predicted effort needed to obtain the rewards; an effect called “effort discounting” [54]. Delay and effort discounting are typically measured in choice tasks, providing the relative impact of costs on reward in decision-making. Previously, we measured the discounting effect of these costs on outcome value by quantifying the relation between the amount of expected cost and the change in operant, reward-directed behavior [55].” (line 263-271)

*(5)     About the relationship between delay and workload discounting, I have naïve questions. First, can we directly compare the two in a paradigm? And how are those two concepts distinguished in monkeys?*
R: In our paradigm, we implemented workload as the number of simple instrumental trials remaining before reward. Usually, as workload increases, the time duration to achieve reward increases. Thus, part of the workload-discounting could somehow be interpreted in terms of delay-discounting. As now explained in the Results section, at the beginning of the paragraph presenting the influence of DAR on cost-processing, we designed the work/delay task to address this issue: in this task the time to reward was matched in the two trial types, allowing us to infer the relative contribution of delay vs physical effort in workload trials: since the time to delay was matched between the two trial types, any effect on motivation that was specifically observed in the workload trials could reliably be interpreted in terms of physical effort.

That approach (the task and the corresponding interpretation) was established in one of our previous studies (Minamimoto et al., PLoS One 2012). In that study, we tested 8 macaque monkeys with this task and showed that half of the monkeys showed significantly higher error rates in workload trials than those in delay trials, in line with the idea that workload could include a significant effort discounting component over and above the delay discounting component, due to the fact that any action takes time. There are many factors that could account for the inter-individual sensitivity to the effort component, as well as the fact that only half of the monkeys showed a significant influence of effort (difference between workload and delay discounting), including genetic or life-history traits.

In the present study, we showed that D2R antagonism selectively increased the sensitivity for workload (i.e., workload-discounting) consistently across monkeys. Thus, our results not only confirm that workload involves a specific motivational process (effort) that cannot be simply interpreted in terms of delay-discounting, but that it provides a potential neurobiological substrate for that distinction.

*(6)     The circuitry changes due to the D1R and D2R blocks are discussed in the Discussion. Of course, there are many difficulties with circuit changes, but is there anything added from the changes seen in PET that we can add to the Discussion?*

R: Unfortunately no. Systemic administered DA antagonist acts uniformly on DAR throughout the brain, but we could not detect changes in brain site-specific effects. In fact, it was evident in our PET imaging results showing that, under pre-treatment condition, the same proportion of specific binding was reduced across different brain areas irrespective of the baseline DAR densities (S1 Fig). Therefore, network change due to D1R/D2R blocks cannot be detected by the changes seen in PET imaging. We discussed this limitation in the final part of the Discussion:

“Second, because of applying systemic antagonist administration, the current study could not determine which brain area(s) is responsible for antagonist-induced alterations of benefit- and cost-based motivation. While our data support the notion that differential neural networks involve workload- and delay-discounting, further study (e.g., local infusion of DA antagonist) is needed to identify the locus of the effects, generalizing our findings to unravel the circuit and molecular mechanism of motivation.” (line 516-521)

***Reviewer #2****: The authors present a pharmacological study addressing the roles of D1 and D2 receptor stimulation on how monkeys process reward incentives and discount effort or waiting time while they do variations of a task to collect rewards. The authors tested the animals either after receiving saline or different doses of SCH23390 to test the involvement of D1 receptors. For the D2 receptors, they administered a fixed dose of haloperidol and tested the animals on different days over a period of 8 days. This was done instead of administering different doses because haloperidol has a high half-life and stays long enough in the system to be able to test the animals with different effective doses.

What appears to be the key feature of the study is that the authors used PET to quantify the receptor occupancy achieved by the doses used in the behavioral experiments. Furthermore, they tested the behavioral impact of different levels of occupancy, effectively doing a dose/response curve for blockade of D1 and D2 receptors.

These are definitely valuable data on a hot topic in systems neurosciences. The question of the relative contribution of D1 and D2 receptors to the dopamine modulation of incentive processing and motivation is timely, and I think that the paper has potential to contribute to clarifying it. However, the manuscript in its current form is far from satisfactory. The presentation is rather poor and requires lots of suppositions from the reader. Thus, there are some aspects when I am not sure if the current analyses are well grounded. Yet in some instances, the authors make unwarranted claims.*

R: Thank you for the positive and constructive comments. According to the advice from the reviewer, we revised the Results section so that, in our opinion, the reader can follow easily. We also provided additional data to support our claims.

(1)*- Presentation - even though the details of the methods are given after the results section, it should be possible to know what the authors did while reading the results section. Details such as the number of monkeys for each experiment should be stated straight away before giving any specific results^1)^. Similarly, the authors should state: that the monkeys are not the same for the PET and the pharmacological experiments^2)^; the gist of the PET analysis (at least name the method used for analysis; whether the analysis is ROI or voxel-based, which ROIs do the results refer to); whether the quantification of receptor occupancy for SCH23390 was done in different sessions;^3)^ how many (or range of) months of training^4)^; the models that are tested to ultimately infer the parameter a in the first experiment should be spelled out so that it is possible to know what the authors are testing before looking at the supplement;^5)^ define what is meant by moderate dose and high dose in the workload and time discounting study and how these doses were derived;^6)^ and that the reward size in the discounting study is constant^7)^.*

R: Thanks for the constructive advice. We added some details in Results, as follows:

1 and 2) Details regarding the number of monkeys and that they are not the same as those for PET

“We performed PET imaging with selective radioligands for D_1_R ([^11^C]SCH23390) and D_2_R ([^11^C]raclopride) in a total of 4 monkeys (3 for each) under awake condition for both baseline (without drug administration) and following antagonist administration.”(line 85-88)

“We next quantified the effects of DAR blockade on behavior using a total of three monkeys not used in the PET occupancy study (monkeys KT, ST and MP; the former two for incentive and all three for cost-based motivation, respectively).” (line 125-127)

3) The gist of the PET analysis (quantification methods, VOI-base, the way of D1 estimate)

“We quantified specific radioligand binding using a simplified reference tissue model with the cerebellum as reference region.

For D_1_R measurement, high radiotracer binding was seen in the striatum at baseline condition (Fig 1A, Baseline). PET scans were obtained after pre-treatment with non-radiolabeled SCH23390 for D_1_R antagonist at different doses (10, 30, 50, and 100 µg/kg), demonstrating that specific tracer binding was diminished in a dose-dependent manner (Fig 1A). We performed a volume-of-interest (VOI)-based analysis quantifying the reduction of specific bindings from baseline, which was homogenous across several brain regions within a blocking condition (S1 Fig). We defined receptor occupancy as the degree of reduction of specific binding using the values from striatal VOI, since they appeared to be the most reliable (see Methods) [40].” (line 88-97)

4) Training period

“All monkeys had been trained to perform basic color discrimination trials in the cued multi-trial reward schedule task [43] for more than 3 months.” (line 142-144)

5) Models

“To quantify the increases in refusal rate, we compared 4 models while considering these two factors as random effects: *model #1*, random effect on *a*; *model #2*, random effect on *a* with fixed *e*; *model #3*, fixed *a* with random effect on *e*; *model #4*, random effect on both *a* and *e* (see S1 Table).” (line 173-176)

6) MO and HO

“To compare the effect of D_1_R vs D_2_R antagonism on cost sensitivity at the same degree of receptor blockade, we assessed the performance of the monkeys under two comparable levels of DAR occupancy for D_1_R and D_2_R at about 50% (called “moderate occupancy” or MO) and 80% (“high occupancy” or HO), and under baseline condition (non-treatment) as control. According to the occupancy study (Fig 1), MO and HO conditions corresponded to pre-treatment with 30 and 100 μg/kg of SCH23390 for D_1_R, and 1 day after and the day of haloperidol treatment for D_2_R, respectively. ” (line 316-321)

7) Reward size in work/delay task

“There were two trial types. In the work trials, the monkeys had to perform 0, 1, or 2 additional instrumental actions to obtain a fixed amount of reward, and the cost (workload) scaled with the number of trials to perform.” (line 274-276)

(2)*- It was unclear how the ANOVAs were performed and how the data from the two or three individual monkeys are treated. Given that there are only 2 or 3 monkeys and that it is impossible to make any random effects inference, I would suggest that the authors analyze each individual separately. This is the most honest way to present the results. Conclusions about the effects of the drugs on monkeys in general is not possible, but this does not mean that this data is not very valuable as human studies cannot provide the value occupancy information and test the range of doses. However, the data needs to be interpreted rightly. Furthermore, ANOVAs need to be followed up with post hoc tests in order to be able to interpret interactions.*

R: Thanks again for the constructive advice. We presented the behavioral data individually and analyses have been conducted accordingly. We agree that two-way ANOVAs may not be helpful if interactive effect is significant, preventing straightforward interpretation of the results. We reviewed all statistical analyses to improve clarity by considering individual variability. For analyzing/estimating the impact of DAR treatment in both tasks (cf. Figs. 3 and 5), we applied a linear mixed model (LMM) analysis for the refusal rates of each individual subject separately. We also examined the relationship between RT and refusal rate individually, and found a consistent property across subjects (Figs 4, 6 and S5 Fig.). We performed ANOVAs and post-hoc tests on the parameters obtained from three subjects (Fig. 5 and 7), allowing us to correctly interpret the DAR blocking effects on discounting parameters. We also performed ANOVAs on other data such as reaction time (S3 Fig) and sucrose preference (S4 Fig) to examine each individual separately, and reported the results of post-hoc tests if needed.

(3)*- Related to the previous point, I do not believe that the authors can say that the effects of D1 antagonism are quadratic when they have evidence for a quadratic effect in one monkey and evidence for saturating monotonic effects in the other monkey. I would only dear to make inferences when the effects are consistent across tested monkeys. And even in this case, inference will need to be tentative as far as random effects cannot be tested.^1)^  Similarly, the authors overinterpret the mean effects of combining antagonists as the effects do not seem to be consistent across monkeys^2)^.*

R: 1) We found a similar tendency of the D1 antagonism effects on motivation where the value of the parameter *a* became smallest at middle doses (30 or 50 µg/kg), instead of the largest one (100 ug/kg). We updated Figure 3c by using locally weighted smoothing (LOESS), so that the quadratic effect was more clearly visible across two monkeys. Still, as the reviewer pointed out, these results were obtained from a limited number of monkeys and therefore we carefully reported this as “a tendency” instead of drawing strong conclusions.

“In both D_1_R and D_2_R blockades, *a* declined according to the increase in occupancy; it gradually declined as D_1_R occupancy increased, but then increased at the highest occupancy, presenting a U-shaped tendency, whereas it steeply declined until 20% D_2_R occupancy, and then continued to decrease slightly until 80% occupancy (Fig 3C).” (line 201-204).

“Moreover, the relationship between occupancy and incentive impact was monotonous for D_2_R, but tended to be U-shaped for D_1_R.” (line 430-432).

2) For the combination of D1R and D2R antagonist treatment, we performed a multiple linear regression analysis with random effect of subjects, similarly as performed for the data under either D1R or D2R antagonism treatment. The effect of treatment on the discounting parameters, *kd* and *Kw*, were further compared using one-way ANOVA followed by post-hoc multiple comparison tests. We found a significant difference in delay-discounting parameter *kd* between control and D1R+D2R treatment, and reported it as “additive effect”. For workload discounting, while the parameter *kw* under D2R treatment was significantly higher than that in control and D1R, this difference disappeared after simultaneous D1R and D2R treatment. We reported the statistical results with interpretation as a trend, as follows:

“When we treated the monkeys with SCH23390 (30 μg/kg) on the day following that of haloperidol injection (i.e., both D_1_R and D_2_R assumed to be occupied at ~50%), the monkeys had higher refusal rates in delay trials than control (Fig 7A, D_1_R+D_2_R block), such that discounting factor (*k_d_*) became significantly higher than in control conditions (*p* < 0.05, Tukey HSD test; Fig 7B, delay). By contrast, this simultaneous D_1_R and D_2_R blockade appeared to attenuate the effect of D_2_R antagonism on workload: the refusal rates in work trials were not as high as in D_2_R blockade alone (Fig 7A), and the difference in workload-discounting factor (*k_w_*) between treated and control or baseline conditions disappeared (*p* > 0.05; Fig 7B, workload). A similar tendency of counterbalancing influence was also seen in the motivation for minimum cost trials (*E_0_*) (Fig 7B). These results suggest that blocking both receptor subtypes tends to induce a synergistic effect on delay-discounting, while their effects on workload-discounting cancel each other out.” (line 354-366)

(4)*- Whereas the estimates of occupancy based on administered dose of SCH23390 appears to be very robust, the estimates of occupancy for haloperidol is much more uncertain, provably reflecting individual differences in the metabolism and elimination of haloperidol. This is important given that occupancy was not measured for the animals that took part in the behavioral experiments. This limitation should be acknowledged and discussed.*

R: We acknowledge that our D2R occupancy data suggested a quantitative limitation to some extent. However, the time courses of behavior and occupancy that recovered from a single dose of haloperidol were consistent, and therefore the conclusion would not change. This limitation is acknowledged and discussed in “*Limitations of the current study*” section in Discussion.

“First, there were relatively larger individual disparities in estimated values of D_2_R occupancy by haloperidol in three monkeys (cf. Fig 1D), which could reflect individual variance of haloperidol metabolism and/or elimination. However, the time course of the recovery of D_2_R occupancy was relatively consistent across subjects, being in line with that of behavioral change.” (Line 507-511)

(5)*- At multiple points in the manuscript the authors present their ideas and results without enough consideration of previous evidence. Some examples: the authors omit a paper by Gan et al (corrected) in Nature Neuroscience^1)^ providing evidence that dopamine neurons are not sensitive to effort costs; Husain's lab in Oxford have some nice studies quantifying the effects of effort discounting and its modulation by dopamine^2)^; Berke's lab in Seattle have provided very important insights on the differential roles of dopamine in learning and performance^3)^.*

R: We appreciate the reviewer’s suggestion, and all these important references have now been included in the Introduction and/or Discussion accordingly.

1) Gan et al., 2010 Nature Neuroscience [30]: in the Introduction

“On the other hand, since DA activity shows little sensitivity to information about effort when it is decoupled from reward, it has been proposed that DA is strongly involved in adjusting motivation based on expected benefits (reward availability) rather than on expected energetic costs (effort) [9, 30, 31].” (line 46-49)

2) Chong et al., Cortex 2015 [18] (Husain’s lab): in the Introduction

“In addition, several studies demonstrated that DA neurotransmission was causally involved in regulation of behavior based on expected costs and benefits [12-18]” (line 36-37)

3) Berke 2018, Nature Neuroscience [17] (Berke’s lab): in the Introduction and Discussion

“In addition, several studies demonstrated that DA neurotransmission was causally involved in regulation of behavior based on expected costs and benefits [12-18]” (line 36-37)

“To understand the role of DA in motivation, it is critical to identify not only the pattern of DA activity and release across costs and benefits, but also the action of DA on DARs [17]” (line 52-53)

“This is in apparent contrast to neurophysiological and voltammetry studies that show a lack of sensitivity of DA release to effort, but comforts the idea that DA function requires the integration of receptor action on top of neuronal activity and releasing patterns [10, 17].” (line 485-488)

(6)*- What do the authors mean by "However, the previous studies did not address the quantitative effect of DAR blockade on incentive motivation; more specifically, there was a lack of experimental data to model the causal relationship among DAR stimulation, reward, and motivation." Certainly, there are previous studies showing that D1 and D2 blockade affects incentive motivation.*
R: This sentence has been rephrased as follows to make it clearer:

“However, the previous studies did not measure the effect of DAR blockade on incentive motivation in multiple rewarding conditions, and therefore data describing the quantitative relationship among DAR stimulation, reward, and motivation is not available.” (line 405-408)

(7)*- Michael Frank models of the direct and indirect pathway suggest a division of labor between D1 and D2 receptor whereby D1 and D2 affect learning from positive and negative reward prediction errors, respectively, but also have different impacts on choice performance and motivation. The latter being strongly controlled by D2 receptors.  See, for instance, the excellent review by Collins and Frank in Psychological Reviews. The authors should discuss this model and how it plays on their results.*
R: We appreciate the reviewer’s important suggestion. We refer to the model in the Discussion:

“Interestingly, a neural network model has been proposed by considering these opposing DA functions of direct/indirect circuit embedded in reinforcement learning framework, successfully explaining the enhancement of effort cost due to D_2_R blockade [79]. This scenario might also explain our finding of a synergistic effect of simultaneous D_1_R and D_2_R blockade on delay-discounting.” (line 498-501)

(8)*- on page 12, how can you rule out that the effects are not related to cognitive but motor impairment? Related to that, in the sentence, "Thus, the effect of D2 manipulation on value-based decision was relatively independent from the effects on cognitive or motor speed itself" is not appropriate. Motor speed is what is measured in the experiment, cognition or motor impairments are hypothesized mechanisms. They should not be mixed up and equated in one and the same sentence.*

R: We agree with the reviewer that these notions were confusing, and we believe that this issue is very similar to the one raised by reviewer #1 (second point). In short, in our frame, we assume that DAR manipulations affect a single process, motivation, which affects several behavioral measures, including RT and refusal rate. Thus, we conducted a new analysis to examine the effect of treatment on the relationship between refusal rate and reaction time (Fig. 4). We found that they were correlated and that there was no evidence that the treatments affect motor and motivational functioning separately. Rather, it seems more parsimonious to interpret that the treatment influenced a single process (i.e., motivation), which governs both decision (refusal rate) and response speed (RT). We revised the interpretation of the reaction time data accordingly as follows:

“For D_1_R blockade, RTs were increased according to the antagonist dose (2-way ANOVA, main effect of treatment, *p* < 1.0 x 10^-13^ for both monkeys; e.g., S3A-C Fig, see details in legend). D_1_R antagonism also tended to increase the proportion of late release (2-way ANOVA, main effect of treatment, *p* = 0.08, monkey KN; *p* = 0.0038 monkey ST; e.g., S3D Fig). A simple account of these effects of D_1_R manipulation on RT is that the modulations in RT across conditions are caused by changes in motivation, such that the positive impact of reward on behavior affects both *whether* monkeys perform the action (refusal rate) as well as *how quickly* they will respond (RT). We reasoned that, if this were the case, then the intersession variability in RT and refusal rate should be correlated. A session-by-session analysis revealed that there was indeed a significant linear relationship between refusal rates and RTs in both monkeys, even when the treatment conditions were changed (Fig 4A; S2 Table). D_2_R blockade also prolonged RTs (main effect of treatment, *p* < 1.0 x 10^-4^; e.g., S3E-G Fig). D_2_R blockade did not change the refusal patterns (i.e., too early or late release) (2-way ANOVA, treatment, *p* = 0.31; e.g., S3H Fig). As with the case of D_1_, there was a linear relationship between refusal rates and RTs across D_2_R antagonism sessions, in which treatment had no discernible effect on the steepness of the slope (Fig 4B; S2 Table). Collectively, these results indicate that refusal rate and RT were similarly affected by DAR manipulation, in line with the concept that DAR affects a central process (motivation), which controls the influence of expected reward on both action selection and execution.” (line 220-237).

(9)*- On page 29, the authors state that 3 monkeys, whose codes are provided, were used for the behavioral experiments. This seem to apply to the discounting experiment but according to the results section and figures, 2 different monkeys were used for the incentive experiment.*

R: Two of three monkeys (ST and KN) were tested for incentive effect, while all three monkeys were tested for delay/workload effects. We are sorry that this was not clear enough. And in line with the advice from Rev #1, we now provide further explanation in the Results to help the reader to understand the experimental conditions.

“We next quantified the effects of DAR blockade on behavior using a total of three monkeys not used in the PET occupancy study (monkeys KT, ST and MP; the former two for incentive and all three for cost-based motivation, respectively).” (line 125-127)

(10)*- In figure 1, there seems to be missing some data points. Does it imply that not all doses were tested in all subjects?*
R: True. Three of four doses were examined in each subject. We stated this in the legend of Fig 1.

“Three of four doses were examined in each monkey.” (line 108)

(11)*- In the introduction, for the sentence "Blockade of either D1R or D2R biases animals' choices in tasks manipulating the cost/benefits trade-off. These biases should be spelled out.*

R: We rephrased this sentence by explaining the results of the effect on effort and delay separately as follows:

“For example, in tasks where animals must exert a higher force to obtain a bigger reward, blockade of either D_1_R or D_2_R shifts preferences towards less efforts, thus less rewards, suggesting a role of DA in effort [24-29].” (line 44-46)

“For example, systemic administration of D_1_R or D_2_R antagonist was shown to increase preference for small immediate rewards over larger, delayed rewards [25, 32-34]. Some of these studies, however, have also shown that blockade of D_1_R [34] or D_2_R [33] has no effect on delay cost.” (line 55-58)

(12)*- Why was a head hold device implanted?*
R: We measured in vivo DAR occupancy under awake condition to minimize the effect of anesthesia. Therefore, all the monkeys for PET experiments had implanted a head hold device. We mention this in the Results.

“We performed PET imaging with selective radioligands for D_1_R ([^11^C]SCH23390) and D_2_R ([^11^C]raclopride) in a total of 4 monkeys (3 for each) under awake condition for both baseline (without drug administration) and following antagonist administration.” (line 85-88)

***Reviewer #3****: Overall, this was an interesting and potentially very useful manuscript for the field, focusing on the effects of DA antagonists on several distinct aspects of motivation. The behavioral paradigms are elegant, and the use of monkeys is critical, because most of the animal studies in this area have been with rodents, and monkeys potentially provide a bridge between the rodent and human literatures. Moreover, the receptor occupancy data are a very important addition. As one can see from my comments, this manuscript stimulated a lot of thinking. My specific comments are listed below.*

R: Thank you for the positive and constructive comments.

*Comments:*
(1) *In the first paragraph of the introduction, the authors state "For motivational value computation, the expected value of benefits (i.e., rewards) has a positive influence, while the cost necessary to earn the expected reward has a negative impact and discounts the net value of reward".  Although one sees wording like this in the literature, it is actually misleading. As written, this statement implies that the reward itself is devalued (i.e., "…net value of reward"). However, when considering what is involved in cost/benefit analyses such as this, the net value is actually the relative value of the whole complex activity related to the reinforcer itself plus the instrumental response with all its associated costs. This issue also was evident in the literature on response/reinforcement matching, going back to the 1960s and 70s.  While a reinforcement value parameter was determined from response/reinforcement matching studies of variable interval responding, it was evident to some researchers that this parameter was not the value of the reward per se, but rather, the value of the whole activity involving the instrumental response and the delivery of the reinforcer (i.e., the entire sequence of behavior that includes the response, its associated biases, and the reinforcer; Williams 1988). Indeed, the focus of the present article on the lack of effect of DA antagonism on relative reward value highlights the importance of these subtle distinctions in wording and terminology.*

R: Thank you for pointing out this issue. We agree that the original version of the first paragraph of the Introduction was not clear enough and was misleading. We revised this part, as follows:

“In our daily lives, we routinely determine whether to engage or disengage in an action according to its benefits and costs: the expected value of benefits (i.e., rewards) has a positive influence, while the cost necessary to earn the expected reward (e.g., delay, risk or effort) decreases the impact of reward value [1-3].” (line 30-33)

(2) *On a related note, in rodent studies it has been shown that in parallel experiments that involve intake of and preference for the reinforcers used in costs/benefit procedures, DAergic manipulations did not affect reinforcer intake or preference (Nunes et al. 2013; Yang et al. 2020).  Moreover, studies involving the progressive ratio/chow feeding choice task in rats, which essentially functions as a discounting task involving increasing ratio requirements, it has been shown that D1 and D2 receptor antagonism produce effects that are very different from the effect produced by reinforcer devaluation (Randall et al. 2012, 2014). These findings are consistent with the conclusion of the authors that DA receptor blockade did not produce a general alteration of reinforcement value.*

R: Thank you. We referred to these previous findings (Nunes et al, [57], Yang et al., [58], Randall et al., 59]) to strengthen our conclusion in the Discussion as follows:

“Our finding, a reduction of incentive impact due to DAR antagonism (cf., Fig 3) is in line with the incentive salience theory, that is, DA transmission attributes salience to incentive cue to promote goal-directed action [12]. The lack of effect of DA manipulation on satiety and spontaneous water consumption is consistent with previous studies in rodents [57, 58]. Our results are also compatible with the idea that DA manipulation mainly influences incentive processes (influence of reward on action) but does not cause a general change of reward processing, which includes hedonic processes (evaluation itself, pleasure associated with consuming reward)[59, 60], although further experiments would be necessary to address that point directly [12].” (line 409-417)

(3) *There have been a large number of rodent studies showing that DA D1 antagonism altered decision making based upon physical effort (Cousins et al. 1994; Nowend et al. 2001; Salamone et al. 2002; Sink et al. 2008; Randall et al. 2014; Yohn et al. 2015; Hosking et al. 2015). The authors seem to suggest that these effects may be solely due to actions on incentive motivation, rather than something to do with work load per se.  However, I wonder if there is a possibility that there are species differences; i.e., perhaps the role of direct vs. indirect pathways and hence the effects of D1 antagonism are different in rodents vs. primates.^1)^ Also, the authors should clarify if the implication of their line of thinking is that blunting of incentive motivation leads to an apparent lack of exertion of effort due to a reduced propensity for action, or if something else is going on^2)^.*

R: 1) As the reviewer pointed out, there is a discrepancy in the effect of D1R antagonism on decision-making based on physical cost between our finding and previous studies. We referred to some of the suggested studies in the Introduction to explain how the present study could help to clarify the complex relation between DARs and effort. We also included these references in the Discussion and directly addressed this issue by discussing the potential reasons for this discrepancy.

“For example, reduced willingness to exert physical effort to receive higher reward was similarly found following D_1_R and D_2_R antagonism in many rodent studies [26, 29, 59], while it was observed exclusively by D_2_R antagonism in other studies [24, 67, 68]. This inconsistency may have two reasons. First, previous studies usually investigated the effect of antagonism on D_1_R and D_2_R along with a relative pharmacological concentration (e.g., low and high doses). In the present study, PET-assessed DAR manipulation allowed us to directly compare the behavioral effect between D_1_R and D_2_R with an objective reference, namely occupancy (i.e., ~50% and ~80% occupancy). Second, the exact nature of the cost (effort vs delay) has sometimes been difficult to identify, and effort manipulation is often strongly correlated with reward manipulation (typically when the amount of reward earned is instrumentally related to the amount of effort exerted, see [10]). Here, using a task manipulating forthcoming workload independently from reward value, we demonstrated that blockade of D_2_R, but not D_1_R, increased workload-discounting in an occupancy-dependent manner while maintaining linearity (cf. Fig 5F).” (line 452-464)

2) The present results suggest two possible mechanisms for reduction in motivation due to DAR antagonism: one is a reduction in incentive motivation or increasing delay discounting, causing a reduction in decision variables and slowing motor response, involving both D1R and D2R. The other is leading to an excessive decrease in decision-making to perform workload trials, which is induced by D2R blocking only. We highlighted these two distinct roles of DA in motivation in the Discussion.

“In sharp contrast to incentive motivation and delay discounting, which involve both D_1_R and D_2_R, the following three observations illustrate a unique mechanism of DA action on workload discounting through D_2_R only. First, workload-discounting increased only with D_2_R antagonism (cf. Fig 5F). Second, the occupancy-dependent effect of D_2_R antagonism on the decision—response time relationship was only seen in workload trials (cf. Fig 6). Third, D_1_R and D_2_R had a synergistic effect in the delay-discounting trials, but an antagonistic effect in the workload-discounting trials (cf. Fig 7). These results extend previous studies demonstrating increased effort-discounting by D_2_R blockade [25, 73]. Besides, our observation that blocking D2R increased refusal rates without slowing response (cf. Fig 6B) empathizes the role of DA in effort-based decision-making, and supports the notion that DA activation allows overcoming effort costs [21]. This is in apparent contrast to neurophysiological and voltammetry studies that show a lack of sensitivity of DA release to effort, but comforts the idea that DA function requires the integration of receptor action on top of neuronal activity and releasing patterns [10, 17]” (line 476-488)

(4) *One of the implications of these studies, taken as a whole, is the incentive motivation, exertion of effort, delay discounting, and reward value are dissociable from each other.  This is consistent with much of the published literature, and is touched upon in the discussion, but is worth emphasizing more because it is an important point.  In addition, I would like to see a bit more emphasis on what the authors mean be incentive motivation, in terms of behavior theory.*
R: We tried to reformulate the whole manuscript to clarify the relation between our studies and these various concepts. We provide clearer operational definitions of how this study addresses these issues both in the Introduction and the Results (e.g., definition of distinct types of cost). In the Discussion we emphasized the separable DA actions on reward motivation, as well as on delay discounting and workload discounting.

We also tried to clarify the distinction between incentive processes (the influence of motivation on behavior) and the hedonic processes (which relate to the pleasure potentially associated with consuming the reward). In the Discussion, we now state the following:

“Our finding, a reduction of incentive impact due to DAR antagonism (cf., Fig 3) is in line with the incentive salience theory, that is, DA transmission attributes salience to incentive cue to promote goal-directed action [12]. The lack of effect of DA manipulation on satiety and spontaneous water consumption is consistent with previous studies in rodents [57, 58]. Our results are also compatible with the idea that DA manipulation mainly influences incentive processes (influence of reward on action) but does not cause a general change of reward processing, which includes hedonic processes (evaluation itself, pleasure associated with consuming reward)[59, 60], although further experiments would be necessary to address that point directly [12].” (line 409-416)

*Minor Comments:*
P 4, "DAR blockades" should be "DAR blockade"

R: Corrected. Thank you.

P 16, "following D1 and D2 antagonisms" should be "following D1 and D2 antagonism"
R: Corrected. Thank you.
