# Supplementary material for: D1- and D2-like receptors differentially mediate the effects of dopaminergic transmission on cost–benefit evaluation and motivation in monkeys
Source: PLoS Biol. 2021 Jul 1;19(7):e3001055. doi: 10.1371/journal.pbio.3001055 (PMC8248602; doi:10.1371/journal.pbio.3001055)
Supplement: Attachment — Submitted filename: response0521.docx [file pbio.3001055.s012.docx]

***Reviewer #2:*** *The authors have successfully addressed my comments. The manuscript is much clearer in terms of the novelty of the matched work and delay tasks and the analysis made. There are only some minor issues remaining.*
R: Thank you for the positive and constructive comments.

*I am still not convinced about the quadratic relationship between D1 blockade and incentive motivation based on the data of two monkeys presented and I would suggest that this statement is further tuned down. Beyond saying that the relationship tends to be U-shaped in the discussion, it needs to be clear that the relationship between D1 blockade and refusal rate is inferred solely based on 2 monkeys and that there is no statistics supporting its existence for this behavior at the population level. In line 180-181 the authors could say "went clearly up for monkey KN but less clearly so for monkey ST".*
R: We agreed that our conclusion are based on 2 monkeys without statistical support, and we have included the following sentence in the Discussion.

“Although this U-shaped effect of D1R blockade was inferred solely based on the refusal rate of two monkeys without statistical support at the population level, and was not found in the RT data,…” (line 462-463)

In addition, we followed the reviewer’s advice and revised the sentence as follows:

“…normalized *a* decreased as the dose of SCH23390 was increased to 30 or 50 μg/kg, but then it increased at the highest dose (100 μg/kg) for monkeys KN but less clearly so for monkey ST (Fig 3A, left)” (line 188)

*In new analysis, the authors show that refusal rates and RT are correlated, possibly both reflecting motivation. If this is the case, and the effects of D1 blockade on motivation are quadratic, shouldn't the effects of SCH23390 on RT also be quadratic?*
R: When considering the data averaged across session, blocking D1R affected refusal rate in an inverse U-shape and increased RT monotonically. When considering inter session variances, however, refusal rates and RT was linearly correlated. Together with the comment above, we mentioned this inconsistency of our interpretation in the Discussion.

“Although this U-shaped effect of D1R blockade was inferred solely based on the refusal rate of two monkeys without statistical support at the population level and was not found in the RT data, …” (line 462-463)

*What do the authors have in mind when suggesting (on line 334-335) that the effects of D2 blockade on workload are through a distinct motivational process? I find the dissociation in RT/refusal rate correlations after different levels of D2 blockade very intriguing and I believe it deserved further discussion.*
R: We refer to ‘Distinct motivational process’ as ‘the overcoming effect of D2R-mediated DA activation’. We have discussed this issue.
“…These results extend previous studies demonstrating increased effort-discounting by D_2_R blockade [25, 73]. Besides, our observation that blocking D_2_R increased refusal rates without slowing response (cf. Fig 6B) empathizes the role of DA in effort-based decision-making, and supports the notion that DA activation allows overcoming effort costs [21].” (line 502-504)

To clarify this and make it easier to link it to the discussion, we modified the sentence in the Results as follows: “Thus, D_2_R manipulation had a distinct influence on RT and refusal rate in workload trials, suggesting that it was acting on behavior through a distinct motivational process, such as overcoming effort costs (see Discussion).” (line 346-347)
